# Case Report: DRESS Syndrome Induced by Two Antituberculosis Drugs in an 8-Year-Old Girl

**DOI:** 10.3389/fped.2022.830611

**Published:** 2022-02-24

**Authors:** Vaidotas Urbonas, Dominykas Varnas, Kristina Mociskiene, Violeta Kvedariene, Odilija Rudzeviciene

**Affiliations:** Vilnius University Faculty of Medicine Clinic of Children's Diseases, Vilnius, Lithuania

**Keywords:** dress, drug reaction with eosinophilia and systemic symptoms, pediatric, drug reaction, antituberculosis drugs

## Abstract

DRESS syndrome is defined as drug-induced hypersensitivity syndrome with rash, eosinophilia, and systemic symptoms. This syndrome is mostly associated with anticonvulsants, antibacterial and anti-inflammatory drugs. DRESS syndrome is a rare disease and is more frequently seen in adults. We present the first case report of DRESS syndrome in an 8-year-old girl, after 3 months of treatment with isoniazid and rifampicin. After discontinuation of drugs and a short course of prednisolone the girl recovered. After 5 years of follow-up, she is healthy and has no complaints but patch tests with isoniazid and rifampicin remain positive. The reported case emphasizes the importance of thorough medical history and including drug reactions in differential diagnosis.

## Introduction

Drug Rash with Eosinophilia and Systemic Symptoms (DRESS) syndrome is a severe adverse drug-induced idiosyncratic reaction occurring 2–6 weeks after the beginning of treatment and is characterized by febrile temperature and skin rash accompanied by hematological abnormalities (hypereosinophilia, atypical lymphocytes) and internal organ involvement (hepatitis or other). Diagnosis of this disease is made according to RegiSCAR (European Registry of Severe Cutaneous Adverse Reaction) scoring system (1).

In a recent systematic review of DRESS syndrome in a pediatric population, the authors found 30 different drugs associated with this disease (2), but in this review, the antituberculosis medication isoniazid was not included as a causative agent of DRESS (3, 4).

DRESS syndrome is an uncommon disease especially in children due to the difficulty of correct diagnosis (1, 5). However, recognizing this disease is of particular importance, as the mortality rate is up to 5.4% (6).

We report, to the best of our knowledge, the first case history of an 8-year-old girl with DRESS syndrome induced by isoniazid and rifampicin.

## Case Description

An 8-year-old girl was diagnosed with mediastinal lymph node tuberculosis. She was treated with isoniazid, rifampicin, pyrazinamide, and ethambutol for 2 months (from January 17 to March 17) and then continued only with isoniazid, rifampicin, and pyridoxine. Liver enzymes (ALT, AST) were checked every month and were within normal range.

Three months after the start of the treatment (April 18), the patient developed fever (38°C), abdominal pain, nausea, vomiting, and on the next day—jaundice and pruritic maculopapular rash on the face, neck, and trunk. On April 22, the patient was hospitalized in Vilnius University Children's Hospital.

Physical examination revealed mild fever (37.6°C), generalized maculopapular exanthema with excoriations on the face, neck, and trunk, icteric sclera, and hepatosplenomegaly. The patient suffered from abdominal pain, nausea, and vomiting. Laboratory tests found abnormal liver function (ALT 1,474.8 U/l, AST 1,272.4 U/l), increased total (347.7 μmol/l), direct (241.9 μmol/l) bilirubin, and GGT (207.9 U/l). Blood test indicated mild eosinophilia (0.41 × 10^9^/l; 6.5%). Data of laboratory findings are summarized in Table 1.

**Table 1:** Laboratory values in the course of the disease.

**Date**	**Blood Eosinophilia (x10^**9**^/L; %)**	**ALT (U/L)**	**AST (U/L)**	**GGT (U/L)**	**TB (μmol/L)**	**DB (μmol/L)**
04–22/23 Initial evaluation	0.41; 6.5	1,474.8	1,272.4	207.9	347.7	241.9
04–24 Starting prednisolone	3.21; 31.2	1,234.1	648.3	184.9	308.7	221.1
04–25 Discontinuation of TB drugs	–	–	–	–	–	–
04–27	1.82; 17.2	947.6	469.1	172.6	253.9	181.7
05–06 Before discharge	0.73; 6.8	631.3	363.8	135.5	65.7	41.6

*ALT, alanine transaminase; AST, aspartate transaminase; GGT, gamma glutamyl transpeptidase; TB, total bilirubin; DB, direct bilirubin*.

Alkaline phosphatase, C-reactive protein level, total protein, and albumins were within normal limits. Serological tests for viral infections, including hepatitis A, B, and C, Epstein-Barr virus, and cytomegalus virus were negative. Anti-Parvovirus B19 IgM was negative, anti-Parvovirus B19 IgG—positive. Ultrasound examination found hepatosplenomegaly, enlarged pancreas and regional lymph nodes, and increased gallbladder wall thickness.

Drug hypersensitivity and acute hepatitis were suspected, thus the decision was made to discontinue earlier prescribed drugs. The patient was given prednisolone 1 mg/kg daily, clemastine 1 mg two times per day, and ursodeoxycholic acid 250 mg two times per day. On April 25 eosinophilia dramatically increased to 3.21 × 10^9^/l (31.2%). Given the association of fever, rash, eosinophilia, and hepatitis, the diagnosis of DRESS syndrome was made and anti-tuberculosis drugs were suspected to be the cause of this severe drug reaction.

Oral prednisolone (30 mg/day) was continued for 6 days. Later, the dose was gradually tapered and stopped after 15 days.

Fever (37–38°C) lasted for 7 days, rash gradually resolved after 10 days and liver function started to improve (Table 1). The patient was discharged home after 17 days.

**Figure 1:**
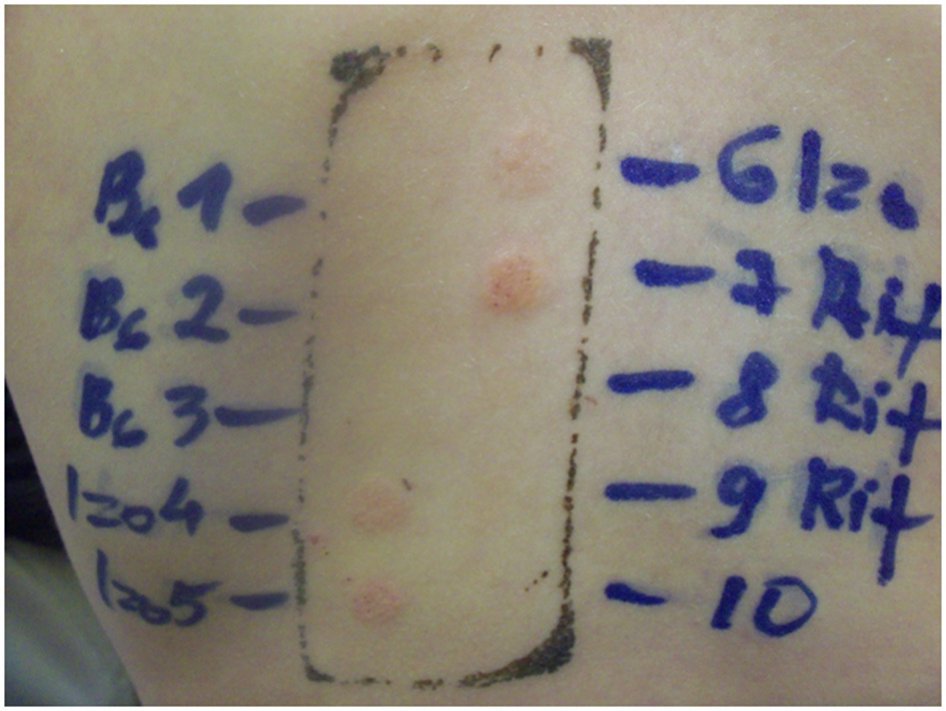
Skin patch test after 72 h.

Two years later the patient was patch tested with culprit drugs. Drug patch tests were performed according to the Guidelines of Contact Dermatitis (7). To avoid any relapse of DRESS in this patient we started the patch tests with a low concentration of drugs (8). Tablets of pyridoxine (50 mg), isoniazid (100 mg), and rifampicin (300 mg) were ground to obtain a homogeneous dilution in petrolatum and water. Patch tests were performed on the upper back area. There were no positive test results at 20 min and no positive reactions with drugs in low dilutions. Positive patch test readings (2+) were evaluated after 48 h for rifampicin diluted to 30% in water and increased to 3+ after 72 h. Another positive patch test (2+) was obtained for isoniazid diluted to 30% in water after 72 h (Figure 1). Isoniazid and rifampicin, but not pyridoxine retained positive readings after 1 week. The diagnosis of DRESS syndrome induced by isoniazid and rifampicin was confirmed. On follow-up, after 5 years the patient was healthy, with normal liver function and an absence of allergies and tuberculosis.

**Figure 2:**
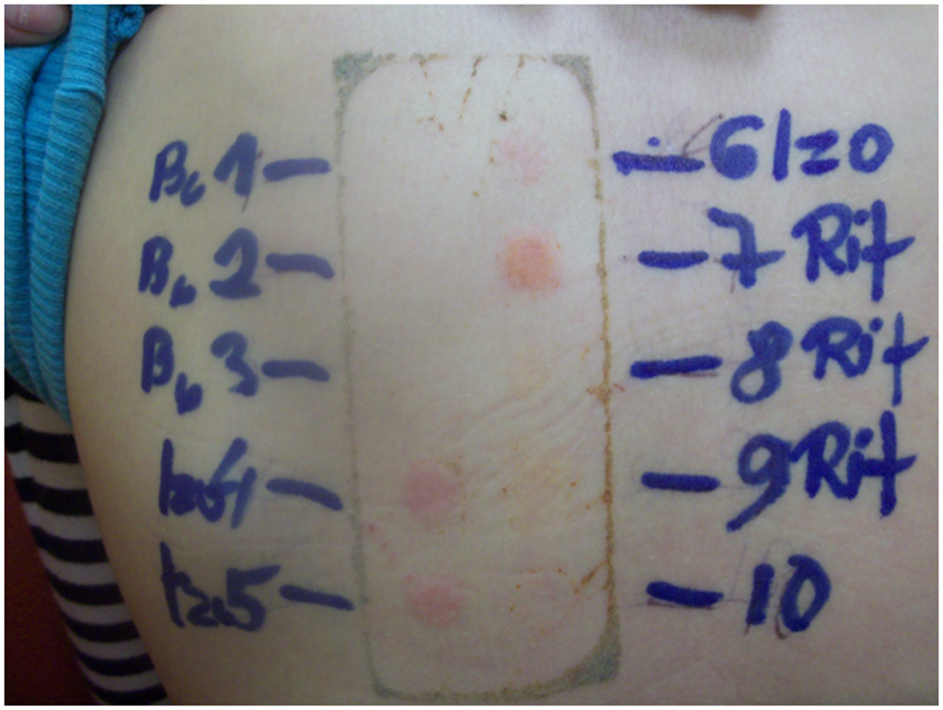
Skin patch test after 168 h. Follow-up after 5 years.

The patch test for the culprit drugs was repeated after 5 years. It was negative after 48 h and 72 h, but after 1 week (168 h) both isoniazid and rifampicin had become positive (2+) (Figure 2).

## Discussion and Conclusion

Skin rash (maculopapular or generalized erythematous) with fever, hypereosinophilia, lymph node enlargement, and the involvement of various organs is characteristic of DRESS syndrome. The liver is the most frequently involved internal organ. Liver lesions (hepatitis, hepatomegaly) are seen in 84.5% of patients, and kidney, lung, central nervous system, heart, and other organs comprise from 1.4 to 20.9% (2).

In our case report, an 8-year-old girl developed DRESS syndrome after 3-months of treatment with antituberculosis drugs isoniazid and rifampicin. The diagnosis of our patient was made according to the RegiSCAR scoring system which has been designed to grade DRESS cases as “excluded,” “possible,” “probable,” or “definite” (1). The patient had definite case because her final score was 6 (fever, enlarged lymph nodes, hypereosinophilia −36%, skin rash > 50% body surface, hepatitis, resolution ≥15 days, negative test results for hepatitis A, B, C, chlamydia, mycoplasma, and blood culture). This is notably a strength of our case report because the diagnosis of DRESS in the pediatric population is difficult given variable presentation and in some literature, the majority of cases were reported as probable/definite (6, 9). Liver lesions occurred from the onset of the disease. In our case, the level of transaminases was elevated >30x above the upper limit of normal (ULN) and gradually started to decrease during the allergy treatment period, but after 20 days of illness still were >10x UNL. After discharge from the hospital patient was regularly monitored in an outpatient setting.

After 5 years of follow up, it was determined that girl is healthy and has a normal liver function.

DRESS syndrome is associated with different drugs; the most frequently reported are carbamazepine, phenytoin, lamotrigine, phenobarbital, and vancomycin (2, 6). Antituberculosis drugs rarely cause this disease and only one pediatric case was previously published (10). The patient was treated with ethambutol, rifampicin, and pyrazinamide (but not with isoniazid). In our case DRESS syndrome developed in the child after 3 months of treatment with the antituberculosis drugs and pyridoxine. The cause of this disease was isoniazid and rifampicin, because during the last treatment month only these medications were used and withdrawal of these drugs, together with prednisolone, cured the patient. Moreover, the diagnosis was confirmed by skin patch tests. The patient also used pyridoxine before the start of the syndrome but the patch test was negative and after 6 months, she consumed pyridoxine in multivitamin form without any complaints. Thus, our patient was not allergic to pyridoxine and this drug could not have caused DRESS syndrome.

In our patient, DRESS syndrome developed after a prolonged period: 12 weeks of the treatment. Usually, the onset of disease starts after around 3 weeks (3, 9). We did not perform an HHV-6 investigation because it did not influence the treatment but infection with HHV-6 during the last month of treatment could explain a long quiescent period with antituberculosis drugs.

In conclusion, this is the first reported case of DRESS syndrome due to a combination of isoniazid and rifampicin and a second case report of DRESS syndrome due to antituberculosis drugs in the pediatric population. It is very important to diagnose this syndrome early so an identified medication could be discontinued and re-exposure avoided in the future. Medical history and knowledge about possible drug reactions is paramount in making the correct clinical decision.

## Data Availability Statement

The original contributions presented in the study are included in the article/Supplementary Material, further inquiries can be directed to the corresponding author/s.

## Ethics Statement

Written informed consent was obtained from the minor(s)' legal guardian/next of kin for the publication of any potentially identifiable images or data included in this article.

## Author Contributions

VU was responsible for making the diagnosis, case report supervision, and wrote the first draft of the manuscript. KM was responsible for daily patient care, follow-up, and case report supervision. VU, KM, VK, and OR were involved in the conception of the paper and critically reviewing the article. DV took responsibility in writing sections of the manuscript, editing the manuscript, and literature review. All authors contributed to manuscript revision, read, and approved the submitted version.

## Funding

This work was supported for publication from Vilnius University.

## Conflict of Interest

The authors declare that the research was conducted in the absence of any commercial or financial relationships that could be construed as a potential conflict of interest.

## Publisher's Note

All claims expressed in this article are solely those of the authors and do not necessarily represent those of their affiliated organizations, or those of the publisher, the editors and the reviewers. Any product that may be evaluated in this article, or claim that may be made by its manufacturer, is not guaranteed or endorsed by the publisher.
